# An *in silico* and *in vitro* approach to elucidate the impact of residues flanking the cleavage scissile bonds of FVIII

**DOI:** 10.1371/journal.pone.0180456

**Published:** 2017-07-06

**Authors:** Behnaz Pezeshkpoor, Ursula Schreck, Arijit Biswas, Julia Driesen, Ann-Cristin Berkemeier, Anna Pavlova, Jens Müller, Johannes Oldenburg

**Affiliations:** 1 Institute of Experimental Hematology and Transfusion Medicine, University of Bonn, Bonn, Germany; 2 Center for Rare Diseases Bonn (ZSEB), University Clinic Bonn, Bonn, Germany; Emory University School of Medicine, UNITED STATES

## Abstract

Coagulation Factor VIII is activated by an ordered limited thrombin proteolysis with different catalytic efficiency at three P1 Arginine residues: Arg^759^> Arg^1708^>Arg^391^, indicating the flanking residues of the latter to be less optimal. This study aimed to investigate, *in silico* and *in vitro*, the impact of possessing hypothetically optimized residues at these three catalytic cleavage sites. The structural impact of the residues flanking Arginine cleavage sites was studied by *in silico* analysis through comparing the cleavage cleft of the native site with a hypothetically optimized sequence at each site. Moreover, recombinant FVIII proteins were prepared by replacing the sequences flanking native thrombin cleavage sites with the proposed cleavage-optimized sequence. FVIII specific activity was determined by assessing the FVIII activity levels in relation to FVIII antigen levels. We further investigated whether thrombin generation could reflect the haemostatic potential of the variants. Our *in silico* results show the impact of the residues directly in the cleavage bond, and their neighboring residues on the insertion efficiency of the loop into the thrombin cleavage cleft. Moreover, the *in vitro* analysis shows that the sequences flanking the Arg^1708^ cleavage site seem to be the most close to optimal residues for achieving the maximal proteolytic activation and profactor activity of FVIII. The residues flanking the scissile bonds of FVIIII affect the cleavage rates and modulate the profactor activation. We were able to provide insights into the mechanisms of the specificity of thrombin for the P1 cleavage sites of FVIII. Thus, the P4-P2´ residues surrounding Arg^1708^ of FVIII have the highest impact on rates of thrombin proteolysis which contributes to thrombin activation of the profactor and eventually to the thrombin generation potential.

## Introduction

Blood clotting factor VIII (FVIII) is a nonenzymatic cofactor of activated factor IX (FIXa). Activated FVIII (FVIIIa) binds on a membrane surface to FIXa and activates factor X (FX) in the tenase complex [1]. The FVIII protein is synthesized as a ~330 kDa single-chain molecule with six distinct domains. It consists of three homologous A-domains, a unique B-domain and two C-domains [2]. Two short acidic segments, a1 and a2, follow the A1 and A2 domains, respectively, and a similar short a3 segment precedes the A3 domain. Due to cleavage at the B-a3 junction, followed by a number of additional cleavages within the B domain, a variably sized heavy chain (A1-a1-A2-a2-B) and a light chain (a3-A3-C1-C2) are generated [3].

FVIII protein is activated by limited proteolysis by either thrombin or FXa [4–6] via cleavage of three peptide bonds at Arg^391^ (a1-A2 junction), Arg^759^ (a2-B junction) and Arg^1708^ (a3-A3 junction) [4]. Thrombin is the physiological activator of FVIII since von Willebrand factor (VWF) inhibits the membrane dependent profactor activation catalyzed by FXa [7]. Upon activation the B domain is released and an active heterotrimer (A1/A2/A3-C1-C2) is formed. FVIII activation occurs in an ordered sequence with an initial cleavage at Arg^759^ followed by cleavages at Arg^1708^ and Arg^391^ [4]. The initial proteolysis at Arg^759^ facilitates subsequent proteolysis at the latter two sites [8], but both proteolysis at Arg^1708^ and Arg^391^ are the critical steps in activation of FVIII via liberating FVIII from the associated VWF [9], and exposing the cryptic functional FIXa-interactive site in the A2 domain, respectively [10].

Previous studies have shown that both thrombin anion binding exosites contribute to the proteolytic activation of FVIII [11, 12], with the binding to the heavy chain being preferred [8, 9]. The biochemical and biophysical characteristics of several thrombin cleavage sites have been studied and analyzed in detail. A theoretical thrombin cleavage-optimized proposed peptide, P(osition)3-P2-P1-P1´-P2´, would exhibit the following amino acid sequence: P2-Pro, P1-Arg, P1´-Ser/Ala/Gly/Thr and P2´-not acidic. Thus the amino acid residues surrounding all three Arg (P1) cleavage sites influence the effectiveness of cleavage by thrombin [13, 14]. Based on this assumption, residues flanking Arg^391^ are considered non-optimal (with only two residues being optimal, P1, P1´) compared to the other two P1 sites that are close to optimal (P2, P1, P1´ and P2´). However, each thrombin substrate and each cleavage site presents its own unique biochemical and biophysical character which defines its cleavage related functional relevance in the overall process. This is also the reason why these cleavage sites might present characteristics which would be normally considered non-optimal, however when considered in the context of a complete process they are fine-tuned according to the spatial and temporal relevance of their specific cleavage. Amongst several known and characterized serine proteases, thrombin stands as a unique example since it has three main factors governing its cleavage efficiency: A) anionic binding exosites, which primarily comprise of surface-exposed residues on thrombin. These residues contribute to binding to the FVIII protein pre-cleavage and therefore provide substrate specificity. B) Residues neighboring the P4-P3-P2-P1-P1´-P2´ residues of Arginine cleavage bonds in FVIII. These residues interact with thrombin and stabilize the reaction intermediate(s) e.g. residues/electropositive atoms contributing to the formation of an oxyanion hole. C) Residues that line up the deep active site cleft of thrombin and contribute to enzyme specificity by their interactions with FVIII residues N and C terminal to the cleavage site. A good deal of atomic knowledge for factor A) i.e. the exosite regions/residues binding to FVIII has been generated [12]. Similar information on factors B) and C) are lacking since crystal/NMR based structure in and around this region in the complex form are lacking. We do not as yet know how thrombin active site cleft adjusts to the three FVIII thrombin cleavage bonds and their adjoining regions. Our present study addresses these lacunae using an *in silico* modeling strategy to first understand the impact of the insertion of each of the native FVIII cleavage bonds and their adjoining regions into the thrombin active site cleft in a comparative and functional sense. Secondly we replace the three thrombin cleavage sites with a hypothetical optimized sequence Leu-Val-Pro-Arg-Gly-Ser (P4-P2´) while keeping the remaining adjoining areas, to further isolate and refine the structure-functional role of each cleavage site residue of the respective cleavage sites. Complementary we performed *in vitro* analysis to evaluate the effect of replacing these residues with the optimized sequence on FVIII activity levels.

In conclusion, our data show that the insertion of the FVIII cleavage bond loops into the thrombin active site cleft is not only dependent on the substrate and enzyme specificity but also on the neighboring residues of the cleavage bonds. Moreover, *in silico* and *in vitro* experiments in context of FVIII activation the residues at Arg^1708^ cleavage play a crucial role.

## Materials and methods

### *In silico* modeling of native FVIII-thrombin cleavage complexes

Since in the crystal structure of FVIII, the thrombin cleavage regions are unresolved or functionally inadequate, we adopted a modeling combined with a simulation based approach. The holoenzyme structures of the thrombin/FVIII cleavage site(s) were generated by designing the cleavage site regions as well as optimized cleavage site regions as *ab initio* modeled peptides (http://zhanglab.ccmb.med.umich.edu/QUARK/; accessed on 11.10.2015–05.12.2015; best scoring model was chosen)[15] and then replacing them on an available factor XIII-(28–37) decapeptide bound to alpha-thrombin complex (PDB ID:1DE7; resolution: 2Å)[16]. The three cleavage sites as well as the three optimized cleavage sites and their neighboring contiguous neighboring residues (30 residues in total) were modeled *ab initio* on the quark server. For the optimized cleavage sites the P4-P2´ residues were exchanged for Leu-Val-Pro-Arg-Gly-Ser (L-V-P-R-G-S) while keeping the adjoining regions the same. To generate the final holoenzyme complex, the factor XIII-(28–37) decapeptide was replaced with FVIII amino acids (18 residues in total) of the three thrombin cleavage site peptide models, one at a time starting with the P1 Arginine residue generating an inserted thrombin cleavage site peptide with the scissile bond in the approximate middle and in enzymatic proximity to the nucleophilic Ser^195^ residue of thrombin.

To resolve the significant clashes within the thrombin active site cleft, each holoenzyme structure was subjected to a 100 ns long simulation (simulation cell with periodic boundaries and 20 Å minimum distances to protein atoms was employed with explicit solvent; force field: Amber03) that involved initial steps of energy minimization by steepest descent to remove conformation stress within the structure, followed by simulated annealing minimization. During these initial steps of energy minimization the distance and geometry between C-α backbone of Ser^195^ (thrombin), Arg-Ser, was fixed. The restraint was removed when the actual simulation started. This allowed the thrombin active site cleft to structurally overcome bumps and clashes while retaining the enzymatic necessity for thrombin cleavage i.e. proximity of Ser^195^ to cleavage site. The structure within the simulation trajectory with the lowest energy was chosen as the holoenzyme model for the particular cleavage site. Additionally, the factor XIII-(28–37) decapeptide was removed from the initial crystal structure and the structure was energy minimized, also with a 100 ns long simulation as described before in order to serve as a reference apoenzyme structure. The final energy minimized holoenzyme structures were inspected with respect to their apoenzymatic conformations to predict the functional implications for each cleavage site insertion.

All structural analysis and simulations were conducted on the YASARA platform and all graphical image rendering and visualization was done with YASARA and Chimera [17]. All structural alignments were made using the MUSTANG function embedded in YASARA. Protein-peptide interface information was extracted for all modeled apoenzymes by submitting them to the PIC (protein interaction calculator) webserver (http://pic.mbu.iisc.ernet.in/; accessed on 5^th^ September 2016) under default conditions of the server.

### *In silico* analysis of the interdependence between cleavage sites Arg^391^ (a1-A2 junction) and Arg^759^ (a2-B junction)

The spatial proximity of Arg^391^ (a1-A2 junction), Arg^759^ (a2-B junction) cleavage sites in the FVIII B domain deleted crystal structure (PDB ID: 2rce; Resolution: 3.7 Å) was used to investigate and present a structural basis for the interdependence of these two cleavage sites. Here we rectified the structural resolution of the regions around these two cleavage sites in the crystal structure. While the Arg^759^ (a2-B junction) cleavage site region is missing in the crystal structure, the region around Arg^391^ (a1-A2 junction) is highly unstructured most likely owing to the lack of almost a 30 amino acid (355–379) residue region that is unresolved and lies just few residues N-terminal to this cleavage site. Therefore, we modeled these regions on the *ab initio* modeling server, Quark (http://zhanglab.ccmb.med.umich.edu/QUARK/; accessed on 02.03.2017–05.03.2017; best scoring model was chosen)(15). The modeled regions were replaced/joined onto the original crystal structure. All heteroatoms were removed from the final structure. The complete structure was subjected to an energy minimizing MD simulation following a similar protocol as followed in the earlier section. The main difference here was that most of the crystal structure atoms were fixed/restrained during the simulation. Only the regions around the two cleavage sites i.e. 50 residues on either side of each cleavage site were left unrestrained for thermal motion. The final structure was chosen as the one with lowest energy post equilibration of the structure during the simulation.

### *In vitro* expression of recombinant FVIII proteins

The native residues harboring the thrombin cleavage sites of FVIII are Q-I-R^391^-S-V-A, E-P-R^759^-S-F-S and S-P-R^1708^-S-F-Q. Exchanges of the residues flanking Arg^391^ (old nomenclature Arg^372^), Arg^759^ (old nomenclature Arg^740^) and Arg^1708^ (old nomenclature Arg^1689^) (P4-P2´) towards the hypothetical optimal sequence L-V-P-R-G-S, universally used as a linker of recombinant fusion proteins, are designated as optimized thrombin cleavage site (O-TCS) 1, O-TCS 2 and O-TCS 3, respectively. Combinatorial constructs are named accordingly (S1 Fig).

Desired nucleotide exchanges in the full-length *F8* cDNA were introduced into the pCI*neo*-FVIII plasmid according to instructions of the iProof High-Fidelity PCR Kit (Bio-Rad Laboratories, Inc., Munich, Germany) using primers listed in S1 Table. Changes were confirmed by DNA direct sequencing of the whole *F8* cDNA. Recombinant FVIII proteins were transiently expressed in COS-1 cells with 4 μg of each plasmid using Lipofectamine™ 2000 (Thermo Fischer Scientific, Darmstadt, Germany) according to the manufacturer´s instructions as previously described [18]. Cells were cultured in Dulbecco’s modified Eagle medium (DMEM, Thermo Fischer Scientific) supplemented with 10% heat inactivated fetal bovine serum (FBS, Thermo Fischer Scientific). Conditioned media was collected 72 hours after transfection.

FVIII activity in conditioned media was assessed on a BCS XP coagulation analyzer using a chromogenic (FVIII:C_chr_) and a clotting (FVIII:C_1st_) assay (Siemens Healthcare Diagnostics Marburg, Germany). FVIII:C levels were normalized to the FVIII wild-type values (FVIII_WT_) in each experiment and mean values and standard deviation were calculated. FVIII antigen (FVIII:Ag) levels in collected media were quantified using the VisuLize™ FVIII Antigen kit (Affinity Biologicals, Ontario, Canada) consisting a polyclonal antibody, according to the manufacturer´s instructions. For all variants the specific activity was calculated as the ratio of the FVIII:C levels measured separately by the chromogenic and one-stage clotting assay to the ELISA based antigen levels (FVIII:C/FVIII:Ag). This ratio was normalized to the same ratio calculated for FVIII_WT_ in each assay and is presented as a percentage of FVIII_WT_. All experiments were performed three times in duplicates.

Thrombin generation assay was monitored by calibrated automated thrombography (CAT) as described by Hemker et al. (17) with a few modifications. Prior to analysis, collected media were supplemented with 15 mM sodium citrate and the FVIII amount was adjusted to 5 ng/ml (to achieve 5 IU/dL FVIII activity) based on measured FVIII:Ag levels. Collected medium was mixed with 4 μM phospholipids (Haemochrom Diagnostica, Essen, Germany) and subsequently diluted 1:1 in FVIII-deficient plasma (Siemens Healthcare Diagnostics). Coagulation was initiated by addition of calcium and thrombin generation was monitored on an automated fluorometer (Fluoroskan Ascent FL, Thermo Fisher Scientific, Weltham, USA) over a time course of 60 min. The thrombograms and four derived parameters (the lag time, time to peak (ttpeak), peak thrombin and endogenous thrombin potential (ETP)), were used for evaluation of the different FVIII variants. Experiments were done in duplicates from three independent transfections.

## Results

### *In silico* analysis of the P1 residues

In order to achieve a comparative view for the insertion of their respective cleavage site residues and a limited number of contiguous neighboring residues within the thrombin active site cleft, we generated six apoenzyme complexes (three native and three optimized peptides). The *ab initio* modeled peptides were in close agreement with the secondary structure predictions made for their corresponding sequences over the PSIPRED server. However, the structural alignment of the holo- and apoenzyme structures (i.e. energy minimized structure of thrombin i.e. PDB ID: 1de7 without any peptide inserted into the thrombin active site cleft) showed differences between the six modeled apoenzyme complexes.

#### *a) The Arg*^*391*^ cleavage site peptide provides the maximum resistance to insertion

**Fig 1:**
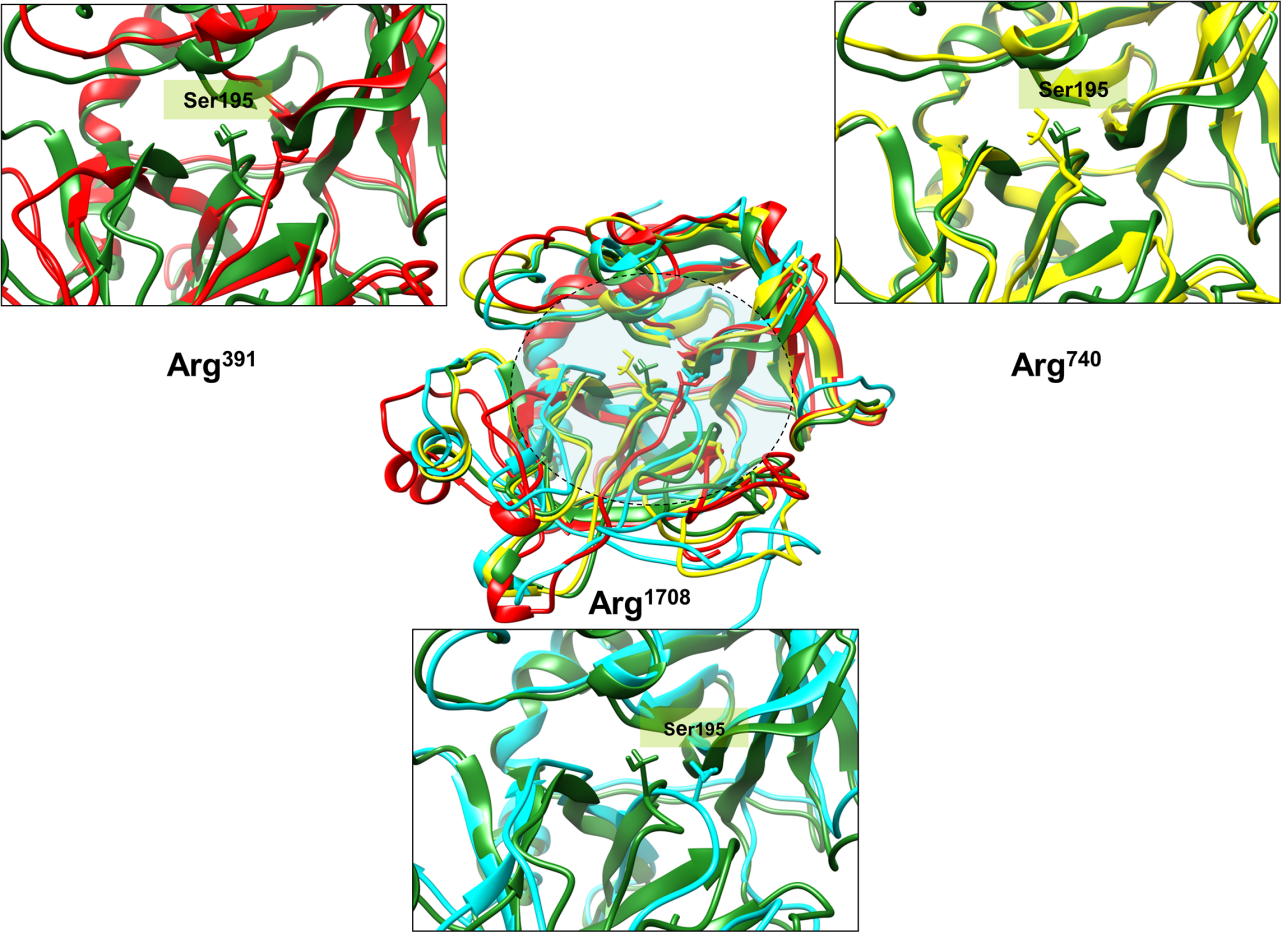
Structural changes in the thrombin active site cleft upon cleavage site peptide insertion. This figure illustrates the differences in structure of the thrombin active site cleft upon the insertion of each cleavage site peptide. The central figure is a multiple alignment of post insertion thrombin structures for all three cleavage sites with the apoenzyme; all of them depicted in ribbon format. Color coding: Green: Apoenzyme, Red: Arg^391^ cleavage site inserted thrombin, Yellow: Arg^759^ cleavage site inserted thrombin, Cyan: Arg^1708^ cleavage site inserted thrombin. The catalytic Ser^195^ thrombin residue is depicted in stick form. The panels surrounding the central inset figure are close up views of each cleavage site inserted thrombin active site cleft structure aligned against the apoenzyme.

**Fig 2:**
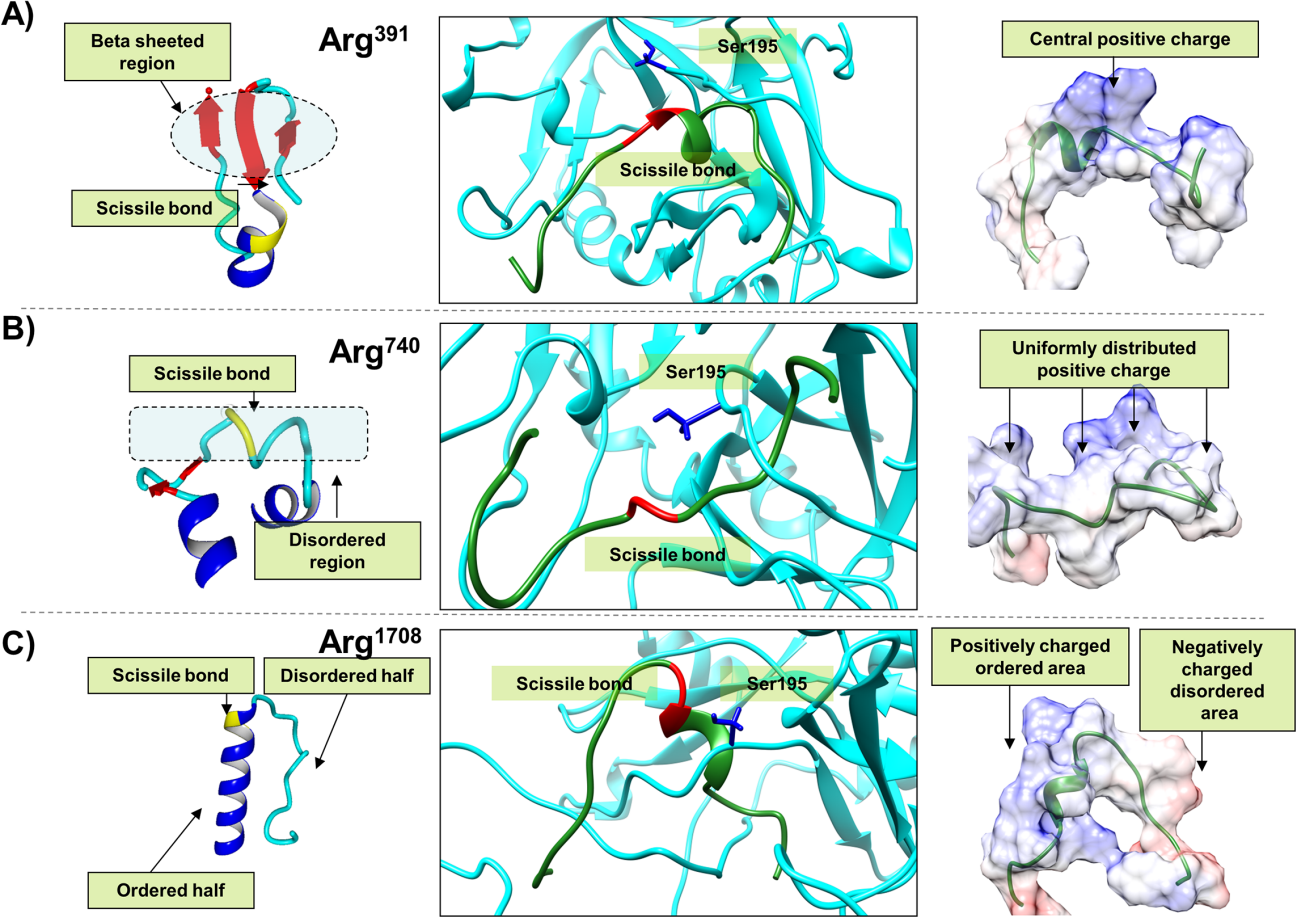
Illustration of the three modeled holoenzyme complexes of the native cleavage sites. This figure illustrates a close up view of modeled holoenzyme complexes for the A) Arg^391^, B) Arg^759^ and C) Arg^1708^ cleavage sites along with the change in the cleavage site peptide structure pre- and post-insertion into thrombin. The central figure in each panel is the close up view of the cleavage site peptide inserted thrombin holoenzyme structure. The backbone depiction is in ribbon format with the thrombin cleavage site FVIII peptide colored green and the thrombin colored cyan. The catalytic Ser^195^ residue is shown in blue stick format. The region on the cleavage site peptide where the scissile bond exists is colored red. The left and the right images in each panel represent the pre- and post-insertion structures of three cleavage site peptides. In the left image i.e. the pre-insertion structures are depicted in ribbon format with coloring as per secondary structure i.e. coil: cyan, helix: blue, beta strand: red. The scissile bond region is colored yellow. The right image is a green colored ribbon format description of the post insertion cleavage site peptide structure. It also shows their molecular surface colored as per their coulombic charges i.e. blue represents positive and red negative charge.

The complex consisting of the Arg^391^ cleavage site peptide appears to undergo the most significant conformational deviation from the holoenzyme (RMSD with holoenzyme: 1.62Å) in comparison to the Arg^759^ (RMSD: 1.08Å) and Arg^1708^ (RMSD: 1.24Å) cleavage site peptide complexes (Fig 1). The Arg^391^ cleavage site pre-insertion appears to be a highly ordered structure consisting of a stable beta sheeted structure comprised of three H-bonded antiparallel beta sheets, including a short helix connecting two of the beta strands (Fig 2A). The cleavage site itself lies between the central beta strand and the connecting helix in this structure. Post-insertion, the helical structure is partly retained however the beta sheeted arrangement is completely lost suggesting a significant change in the secondary structure. The Arg^391^ cleavage site peptide structure shows a number of Lysines close to the scissile bond resulting in an electrostatic positive center that is stabilized by Aspartic acid and Glutamine residues close to the Ser^195^ (Fig 2A).

#### *b) The Arg*^*759*^ cleavage site is provides the least resistance to insertion

The Arg^759^ cleavage site peptide is a largely disordered coil like structure connected by two short helixes at the N and C terminal (Fig 1). Insertion of this peptide does not require significant rearrangement of the secondary structure since even post-insertion it shows a largely disordered structure with the pre-insertion end short-helixes disappearing into random coils (Fig 2B). The Arg^759^ cleavage site peptide consists of positively charged Lysine, Histidine and Arginine residues distributed across its surface (Fig 2B). It is stabilized along its length by positive residues close to the scissile bond or electronegative atoms of residues lying on the surface of the active site cleft.

#### *c) The Arg*^*1708*^ cleavage site post-insertion most likely forms the most stable substrate-enzyme complex

The Arg^1708^ cleavage site peptide pre-insertion has a symmetrically divided structure i.e. half of it is in disordered form and the other half is a highly ordered helix. Interestingly the cleavage site occurs exactly at the helix-coil boundary (Fig 1). Insertion of this peptide involves changes in the ordered helix half of this peptide. However the changes are not as significant as the Arg^391^ cleavage site as only a few intra-helical hydrogen bonds are lost and the helical half partially retains its helical character (Fig 2C). The Arg^1708^ cleavage site peptide harbors positively charged residues in the helix half stabilized by negatively charged Aspartic acid and Glutamine residues close to the thrombin Ser^195^ (Fig 2C). The disordered coil half bears negatively charged residues stabilized by electropositive atoms on the surface of the active site cleft. This site is electrostatically the most satisfied of the three peptides comprised of 53 hydrogen bonds, 5 ionic, 4 cation-pi interactions and one aromatic-sulphur interaction along its length.

#### d) Residue optimization around the P1 residues does not alter the secondary structure

**Fig 3:**
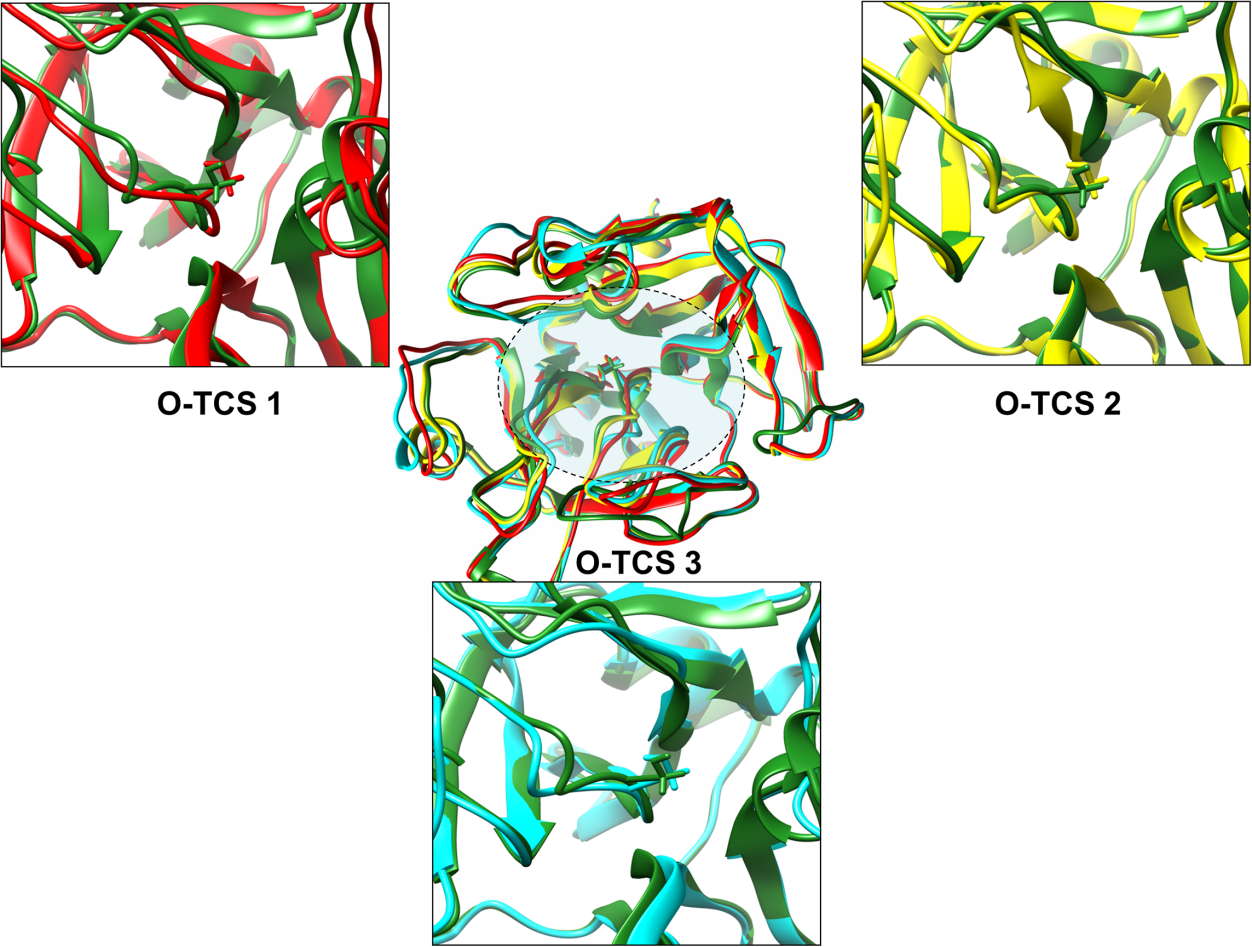
Structural changes in the thrombin active site cleft upon cleavage site regions peptide with consensus cleavage sequence insertion. This figure illustrates the differences in structure of the thrombin active site cleft upon the insertion of each cleavage site peptide with the consensus sequence. The central figure is a multiple alignment of post insertion thrombin structures for all three cleavage sites with the apoenzyme; all of them depicted in ribbon format. Color coding: Green: Apoenzyme, Red: O-TCS 1 cleavage site inserted thrombin, Yellow: O-TCS 2 cleavage site inserted thrombin, Cyan: O-TCS 3 cleavage site inserted thrombin. The catalytic Ser^195^ residue of thrombin is depicted in stick form. The panels surrounding the central inset figure are close up views of each cleavage site inserted thrombin active site cleft structure aligned against the apoenzyme (and therefore of the region specified in the shaded area in the central figure). Depiction code follows that of the central figure.

**Fig 4:**
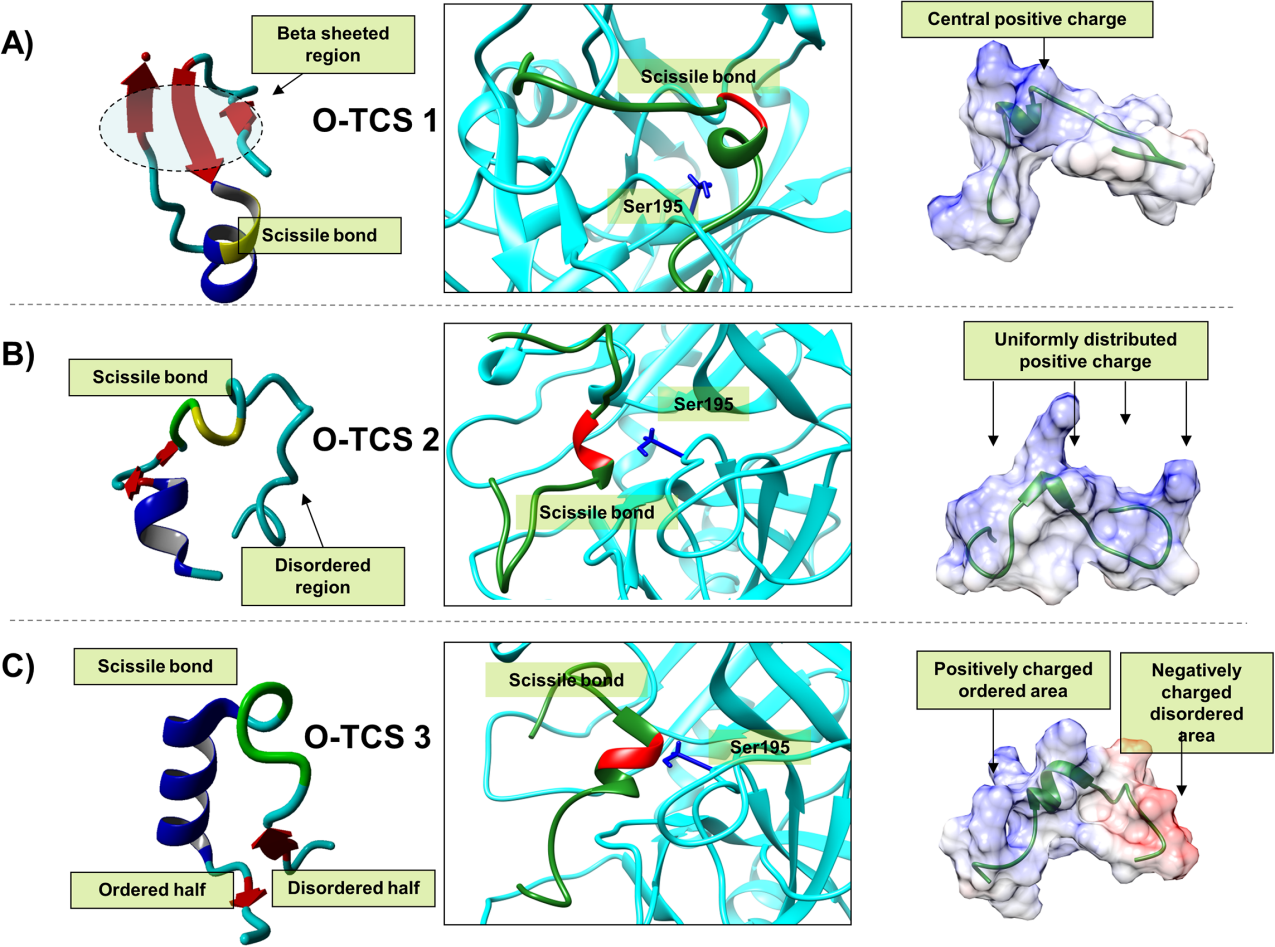
Illustration of the three modeled holoenzyme complexes of the optimized cleavage sites. This figure illustrates a close up view of three modeled holoenzyme complexes (for consensus cleavage site peptides) along with the change in the consensus cleavage site peptide structures pre and post insertion into thrombin. The central figure is the close up view of each consensus cleavage site peptide inserted thrombin holoenzyme structure. The backbone depiction is in ribbon format with the thrombin consensus cleavage site Factor VIII peptide colored green and the thrombin colored cyan. The catalytic Ser^195^ residue is also shown in blue stick format. Also the region on the consensus cleavage site peptide where the scissile bond exists is colored red. The left and the right images corresponding to the central image each represent the pre and post-insertion structures of three consensus cleavage site peptides. The left image i.e. the pre-insertion structures are depicted in ribbon format with coloring as per secondary structure i.e. coil: cyan, helix: blue, beta strand: red. The scissile bond region is colored yellow. The right image is a green colored ribbon format description of the post-insertion consensus cleavage site peptide structure. It also shows their molecular surface colored as per their coulombic charges i.e. blue represents positive and red negative charge.

Before considering the implications of insertion of each optimized cleavage site on the thrombin active site cleft, we investigated the native non-inserted structure of each optimized peptide model. Interestingly, secondary structure wise none of the three optimized peptide models differ from their respective native cleavage site peptide model (Fig 3). Moreover, the secondary structure predictions, based on the optimized sequences, also didn’t suggest significant or marked differences with their original cleavage site sequences. However, the differences become apparent after the process of insertion via simulation and energy minimization is conducted. Post insertion none of the optimized sequences aligns correctly with their native cleavage site models (Fig 4A–4C). Although similar to their native cleavage site models, they end up acquiring primarily disordered flexible forms. As far as the holoenzyme is concerned, similar to the insertion process observed for the native cleavage site peptides, the optimized peptide models also harbor changes mostly on the active site cleft shown in their holoenzyme structure. The maximum conformational change in the holoenzyme observed post-insertion of the optimized sequence was for site O-TCS 3 (RMSD: 1.213Å) followed by a similar value for site O-TCS 1 (RMSD: 1.193Å). The O-TCS 2 site showed post-insertion the lowest degree of conformational change in the holoenzyme (RMS: 0.724Å). This trend is similar to that observed for the respective native cleavage sites.

#### e) Differences in post-insertion interactions bring about functional differences in cleavage of O-TCSs

Detailed inspection of the peptide-thrombin active site cleft interfaces highlight a number of differences between the native cleavage site and the optimized one (Fig 4). These differences most likely translate into differences observed in activities/rate of FVIII activation between optimized and native cleavage sites.

#### f) Interdependence between cleavage sites Arg^391^ and Arg^759^

**Fig 5:**
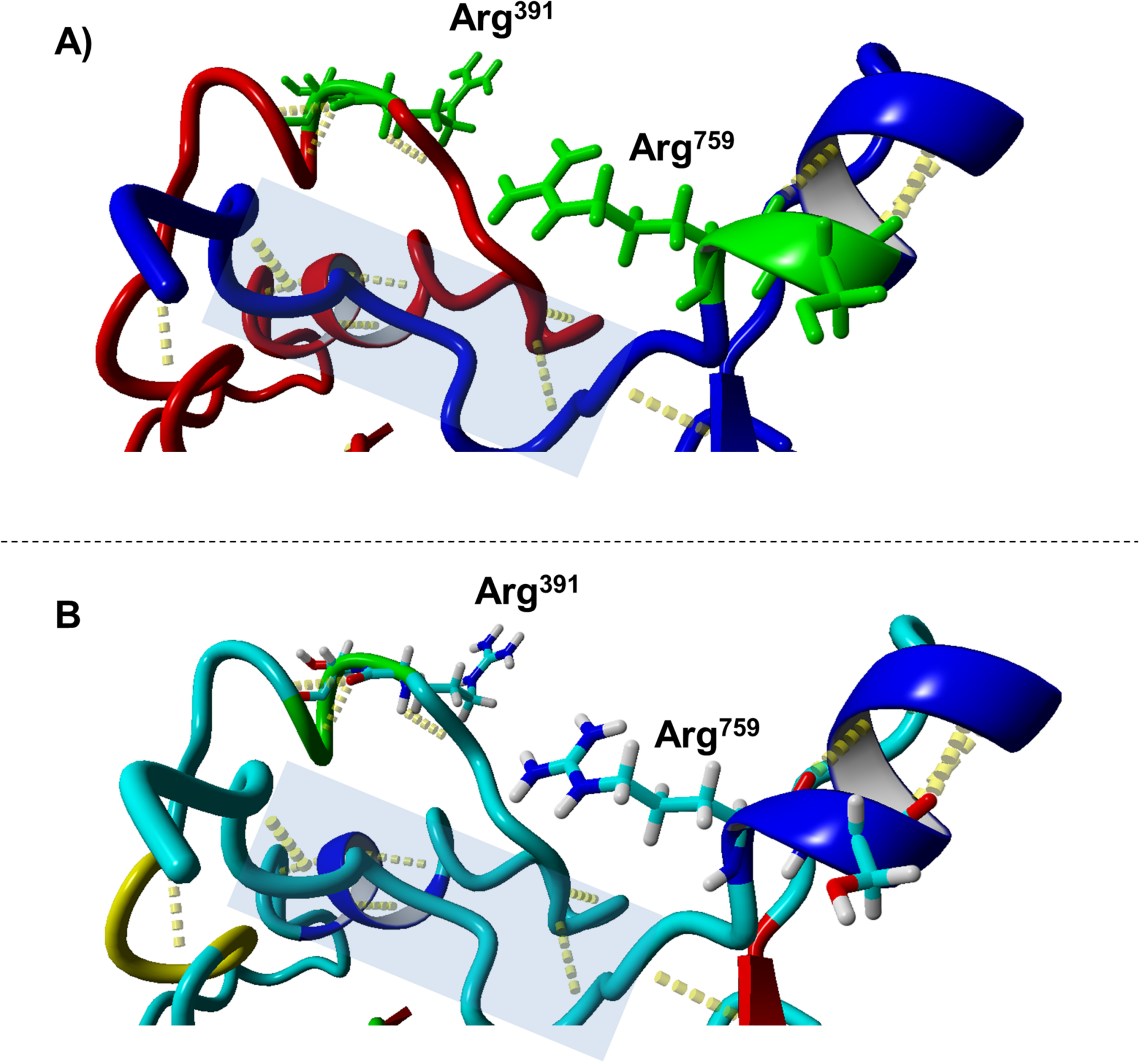
The cleavage site region interface. The panels A and B in this image illustrate a close up view of the Arg^391^ (a1-A2 junction), Arg^759^ (a2-B junction) cleavage site regions and their putative interactions in the crystal structure of FVIII. The cleavage site regions Arg^391^ (a1-A2 junction) and Arg^759^ (a2-B junction) have been remodeled. Both panels show the same view but are colored differently. In both panels the backbone is depicted in ribbon format. Panel A illustrates the region surrounding the Arg^391^ (a1-A2 junction) as a red ribbon while the Arg^759^ (a2-B junction) region is depicted in blue ribbon format. In Panel B both regions are colored based on their secondary structure (i.e. blue: helix, cyan:coil,yellow:short helix, red:beta sheet). The cleavage site P1 and P1´ residues are depicted in stick format. In Panel A they are colored green while in Panel B they are colored based on atom (i.e. white:hydrogen, blue: nitrogen and cyan:carbon). In both panels the hydrogen bonds are depicted as yellow colored dots extending from their donor to the acceptor atom. The interface region between the two cleavage site regions is marked with a blue shaded area.

The final structure with the remodeled Arg^391^ (a1-A2 junction), Arg^759^ (a2-B junction) cleavage sites equilibrated at ~2.0 Å after a period of 12 ns. In this structure, we clearly observe that the two regions carrying the two cleavage sites interact with each other spatially through a series of hydrogen bonded interactions. On a comparative note, the P1 residue of the Arg^759^ (a2-B junction) cleavage site is better exposed (Solvent accessible area: 136.31 Å^2^) than that of the Arg^391^ (a1-A2 junction) cleavage site (Solvent accessible area: 163.82 Å^2^) owing to the conformational fold resulting from interactions between these two regions. The Arg^391^ (a1-A2 junction) cleavage site is clearly better hidden of these two cleavage sites in this structure (Fig 5).

### Comparison of specific activity and TGT of FVIII variants possessing residue optimization

#### a) Single TCS optimization

**Fig 6:**
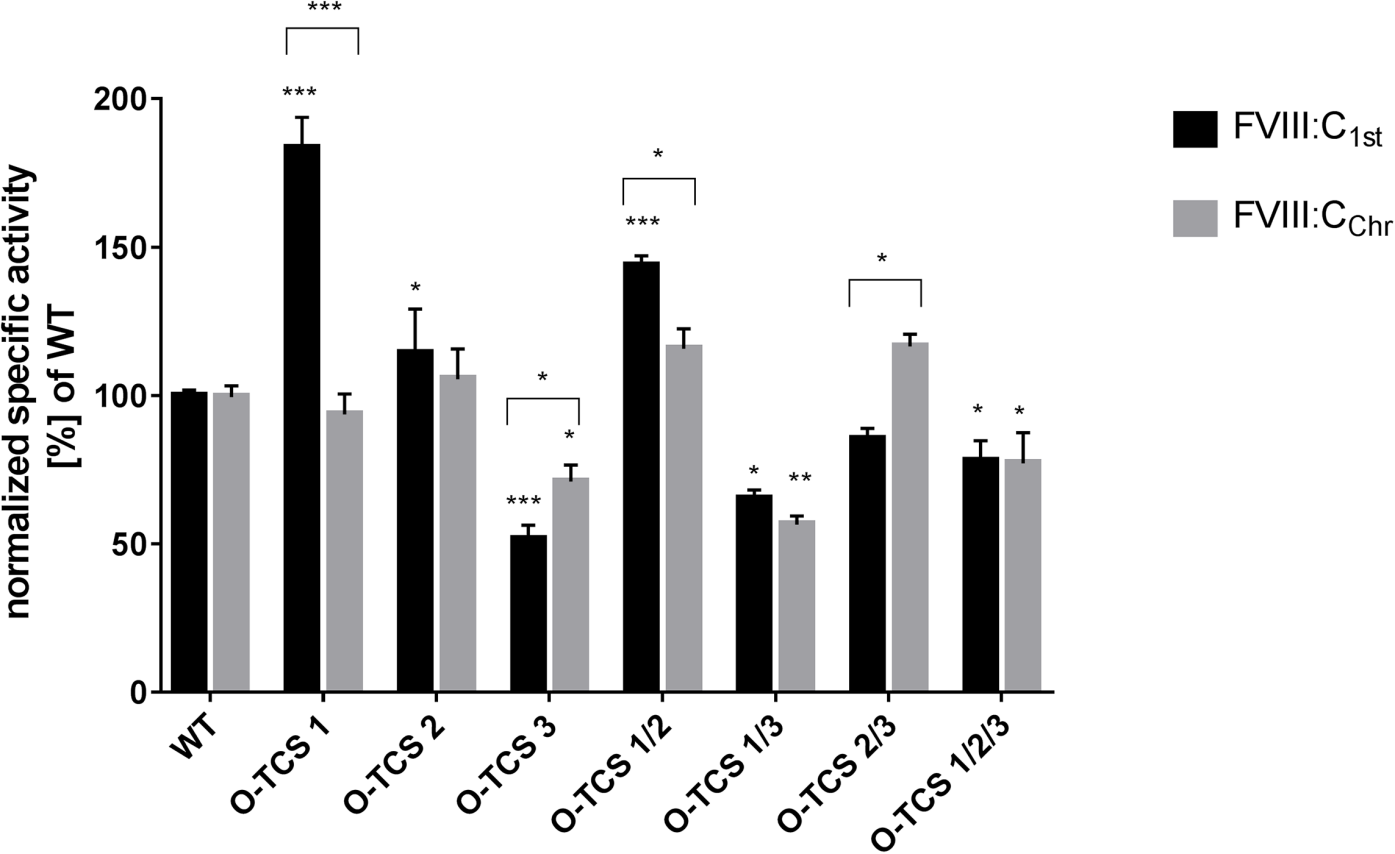
Specific activity of FVIII variants possessing an optimization of sequences flanking the thrombin cleavage site based on FVIII activity levels measured by FVIII:C_1st_ and FVIII:C_chr_. Mean values with standard deviation from three independent experiments in duplicates are shown (* = P ≤ 0.05, ** = P ≤ 0.01, *** = P ≤ 0.001).

For O-TCS 1, according to FVIII:C_chr_ no difference in specific activity was observed when compared to the FVIII_WT_ (Fig 6). Interestingly, the clotting assay showed significant increase of FVIII specific activity (183% of WT in FVIII:C_1st_). A different picture was observed for the O-TCS 2 variant. Here, FVIII:C_chr_ and FVIII:C_1st_ showed slightly higher specific activities compared to the FVIII_WT_ (not significant). Considering the O-TCS 3 variant, positioning of more optimal residues flanking Arg^1708^ resulted in a significant reduction of specific activity obtained in both assays with the clotting assay being more affected than the FVIII:C_chr_ (71% and 52% of WT according to FVIII:C_chr_ and FVIII:C_1st,_ respectively).

#### b) Multiple TCS optimization

For the combinatorial construct O-TCS 1/2, replacing the P4-P2´ residues flanking both Arg^391^ and Arg^759^ with L-V-P-R-G-S, led to an increase of specific activity based on both assays (FVIII:C_1st_ (144% of WT)> FVIII:C_chr_ (115% of WT), Fig 6). Contrary results were observed for the combinatorial construct O-TCS 1/3, where a significant reduction (~50% of WT) of specific activity was observed. For the variant O-TCS 2/3, where sequences flanking the Arg^759^ and Arg^1708^ were optimized, an opposite effect was observed for the chromogenic assay compared to the clotting assay. While the specific activity was slightly increased according to the chromogenic assay (116% of WT), a significant reduction of the value was observed for the clotting assay (85% of WT). When all three native thrombin cleavage sites were optimized, in both assays a significant reduction of specific activity was observed with a similar reduction for the FVIII:C_chr_ and FVIII:C_1st_ (~77% of WT).

#### c) Thrombin generation potential of FVIII variants

**Fig 7:**
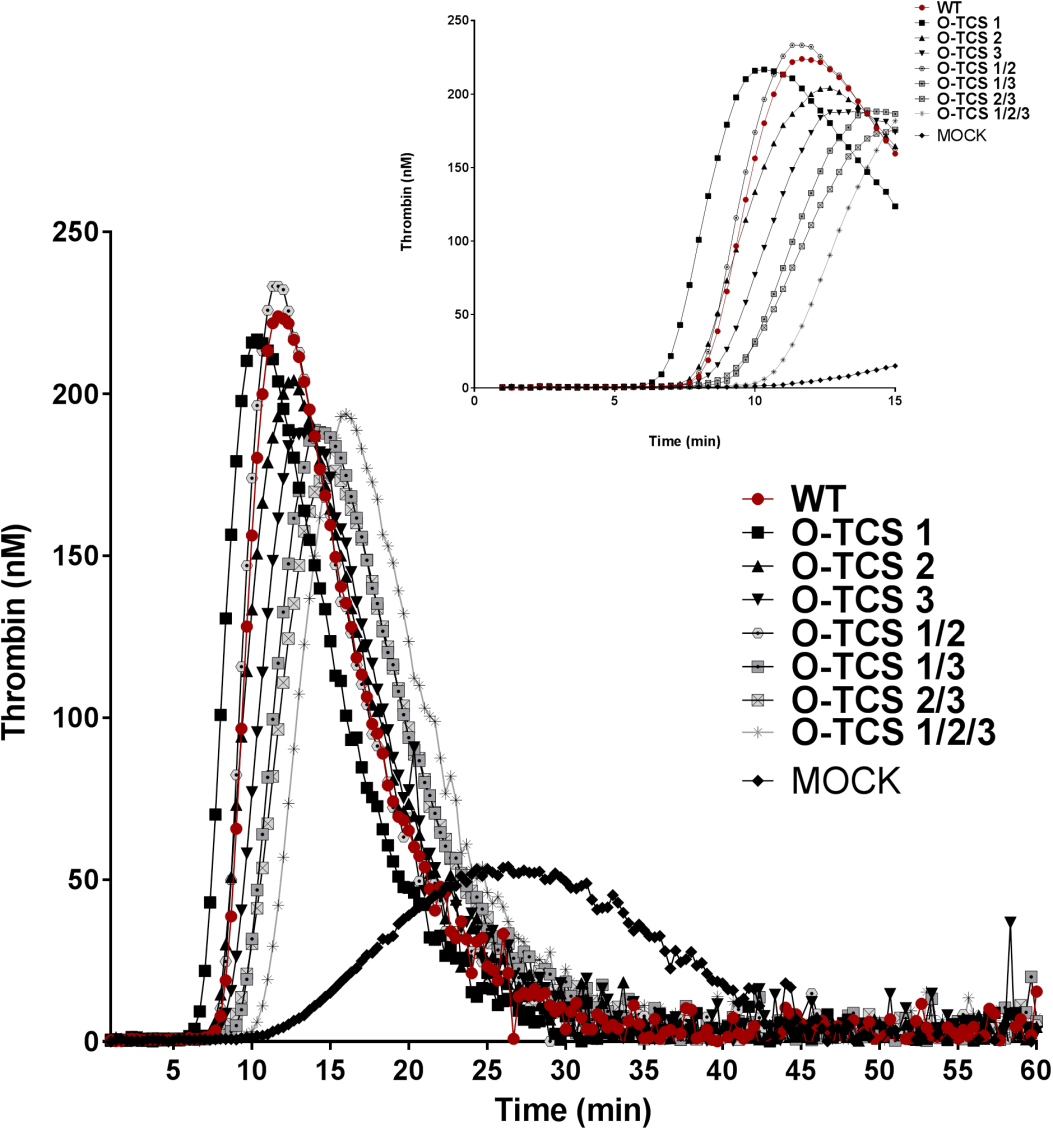
Thrombin generation potential of FVIII variants possessing an optimization of sequences flanking the thrombin cleavage site. FVIII-depleted plasma was reconstituted with FVIII variants and thrombin generation was measured in the absence of TF. All experiments were performed at least three times. Representative thrombin generation curves are shown.

After antigen adjustment, FVIII variants were diluted in FVIII deficient plasma and assessed by a thrombin generation test (Fig 7, Table 1). Thrombin generation of FVIII_WT_ was initiated after 8 min and peak thrombin generation (225 nM) was reached after 11 min and the ETP was 2024 nM*min. The studied variants showed a similar thrombin generation profile compared to the FVIII_WT._ However, changes in the lag-time, peak height and time to peak (ttpeak) reflecting the amplification, propagation, termination phase of the coagulation assay showed slight differences to the FVIII_WT._ The amplification phase of coagulation as reflected by the lag time was increased for the O-TCS 1/2/3 variant (10 min) and decreased for the O-TCS 1. This finding suggests a shorter activation time needed for the O-TCS1 in line with the one-stage assay results. Furthermore, all optimized variants harboring a change at the Arg^1708^ cleavage site exhibited a reduction in the total amount of the generated thrombin (Fig 7, Table 2). The propagation phase of coagulation reflected by the ttpeak value, the time to reach the thrombin peak was increased in all variants except the O-TCS 1 and O-TCS 2 with a major effect for the O-TCS 1/2/3 variant (1.4 fold increase).

**Table 1:** Mean of relative FVIII activity levels (FVIII:C) and FVIII antigen (FVIII:Ag) levels after exchange of native cleavage sites into consensus sequence. The mean is calculated from three independent experiments performed in duplicate. (WT, wild-type; SD, Standard deviation of mean).

Construct	new nomenclature	old nomenclature	FVIII activity and antigen measurements					
			FVIII:C_chr_		FVIII:C_1st_		FVIII:Ag	
			Mean	SD	Mean	SD	Mean	SD
WT	-	-	100,7	2,8	99,5	4,2	100	1,4
O-TCS 1	O-TCS 391	O-TCS 372	43,8	7,4	83,4	5,1	45,7	1,5
O-TCS 2	O-TCS 759	O-TCS 740	89,4	1,5	94,7	4,1	84,4	3,6
O-TCS 3	O-TCS 1708	O-TCS 1689	71,7	5,3	51,1	4,3	99,7	1,3
O-TCS 1/2	O-TCS 391/759	O-TCS 372/740	116,3	8,5	142	4,1	99,2	2
O-TCS 1/3	O-TCS 391/1708	O-TCS 372/1689	64,3	3,1	73,2	4,2	112,6	2,6
O-TCS 2/3	O-TCS 759/1708	O-TCS 740/1689	83,5	7,8	60	4,3	70,4	1,7
O-TCS 1/2/3	O-TCS 391/759/1708	O-TCS 372/740/1689	70,5	10,6	70	6,2	90,9	4,2

**Table 2:** Thrombin generation parameters of FVIII variants. FVIII concentration for each variant was adjusted to 5 ng/ml in complete medium. Subsequently each variant was diluted 1:1 in FVIII deficient plasma and triggered with 4 μM of phospholipids. The mean is calculated from three independent experiments performed in duplicate. (WT: wild-type; ETP: Endogenous thrombin potential; ttpeak: time to peak; SD: Standard deviation of mean).

Construct	Parameters of thrombin generation test							
	Lag time (min)		Peak (nM)		ETP (nM*min)		ttpeak (min)	
	Mean	SD	Mean	SD	Mean	SD	Mean	SD
WT	8	0,2	225	6,8	2024	37.0	11	0,2
O-TCS 1	6	0,12	217	1,1	1929	26,1	11	0,3
O-TCS 2	8	0,17	216	4,4	1995	21,0	11	0,4
O-TCS 3	8	0,08	186	1,3	1871	14,9	13	0
O-TCS 1/2	8	0,04	234	2,3	2023	9,7	12	0,1
O-TCS 1/3	9	0,04	190	1,9	1937	20,6	15	0,1
O-TCS 2/3	9	0,28	179	1,9	1835	27,1	15	0,3
O-TCS 1/2/3	10	0,29	194	6,6	1935	49,9	16	0,2

## Discussion

In this study we investigated the impact of the residues flanking the Arginine cleavage sites in FVIII protein by performing *in silico* and *in vitro* analysis of FVIII variants harboring a hypothetical optimized thrombin cleavage sequence (L-V-P-R-G-S) [13, 14, 19].

Observations from the *in silico* analysis leads us to believe that differences in activity can be attributed to two major factors: A) the quantitative differences in strength for the local secondary/tertiary structural conformations attained by the cleavage sites. B) The nature of the biophysical and biochemical interactions within the apoenzyme complex. The first factor is well demonstrated by the tertiary (the *ab initio* models) and secondary structure predictions of the cleavage site regions which remains the same even when the P4-P2´ residues are replaced with the optimized L-V-P-R-G-S sequence. However, the existence of amino acids with altered secondary structure propensities indicates that the strength of these similar looking structures is different. Their influence is likely of the quantitative in nature. The second factor namely the interactions within the apoenzyme complex determines the stability of the apoenzyme and therefore product turnover and efficiency of cleavage. Irrespective of the initial local structural conformation, the optimized sequences interact differently with the walls of the thrombin active site cleft as well as the regions around the thrombin catalytic triad compared to the native cleavage sites.

The results revealed for the O-TCS 1 variant suggest that positioning of more optimal residues flanking Arg^391^ mitigates the need for initial cleavage at Arg^759^ to facilitate this step [8, 20]. This observation is most likely a combination of the quantitative differences in the structural transition of Arg^391^ cleavage site peptide pre- and post-insertion into the thrombin cleft as well as the interactions taking place post-insertion for this cleavage site. Pre-insertion, this site is the most ordered structure amongst the three peptides while post-insertion it transforms to a random coil structure to facilitate the flexibility of cleavage. Other studies have shown that the Arg^391^ cleavage site is buried and its exposure necessitates the cleavage at the other Arg sites [9]. Substitution of these residues with a consensus sequence bearing residues with lower propensity to occur in buried beta sheets facilitates the structural transition pre- and post-insertion in a positive manner thereby resulting in higher activity.

Moreover, our results demonstrate that in the context of cleavage sites interdependence, the Arg^391^ (a1-A2 junction), Arg^759^ (a2-B junction) are dependent which can be attributed to the mutual spatial interactions of the regions on which they occur. In our models we observed a masking effect on the P1 residue of the Arg^391^ (a1-A2 junction) cleavage site owing to the conformational status of the region that forms the interface between these two cleavage sites. Since Arg^759^ (a2-B junction) would be the first to be cleaved (and naturally so), this will initiate a series of conformational changes. As we have observed for the modeled peptides of the cleavage sites, cleavage by thrombin usually implies the disruption of local secondary structure around the cleavage site in order to accommodate the cleavage site with the thrombin catalytic cleft. Since part of the region that comes to lie within the thrombin catalytic cleft during the cleavage reaction is also part of the interface between the two cleavage sites, naturally this interface will also be disrupted thereby relieving the P1 residue of the Arg^391^ (a1-A2 junction) cleavage site of its masking effect and increasing exposure for the same thereby facilitating the subsequent cleavage of this site as well. This we believe is the structural basis for interdependence between these two sites.

When looking at the residues individually, the native site of Arg^391^ contains three residues (Glutamine, Isoleucine and Valine) with high propensity for buried beta sheet structures [17]. However, the consensus sequence has only one residue (Valine) with a high propensity scale within buried beta sheets. Secondary structure predictions/*ab initio* models do not suggest significant differences in the secondary/tertiary structure between the native Arg^391^ cleavage site and the O-TCS 1 variant. However as mentioned before owing to different amino acid composition of the variant with different secondary structure propensity, quantitative differences in the strength of these structures and their ability to subsequently assume disordered coiled forms within the active site cleft would lead to differences in activity. Additionally the O-TCS 1 apoenzyme complex shows a gain of several hydrogen bonds (main chain, side chain both) (S2 Table) as well as cation-pi interactions over the native cleavage site apoenzyme complex. This suggests a stable substrate enzyme complex accounting for an efficient cleavage at this site.

According to the literature, activation of FVIII by thrombin occurs through proteolysis in an ordered cleavage pattern, Arg^759^ >Arg^1708^ >Arg^391^ with the bond at Arg^759^ also being the fastest to be cleaved [8]. Our *in silico* model reveals very minor differences in secondary structure in the Arg^759^ cleavage site peptide model structure, pre- and post-insertion indicating that the least amount of energy would be required for the insertion of this cleavage site making it the fastest cleaved site amongst the three sites. This is even more evident when the optimized sequence is inserted in the Arg^759^ cleavage site. The conformational change post-insertion for the holoenzyme is <1Å which might account for the slightly higher specific activity observed for this variant compared to the native cleavage site. When the Arg^759^ cleavage site is replaced with the O-TCS 2 variant, it loses a number of hydrogen bonds within the apoenzyme complex (S2 Table). This indicates that although in terms of structure, this site maybe the easiest to insert amongst the remaining three, the peptide-enzyme complex intermediate for the optimized sequence is less stable than the native sequence. *In vitro* expression of the O-TCS 2 variant does not really show a decrease in activity compared with the wild type as might be anticipated with loss of interaction within the substrate-enzyme complex. Instead activity-wise we observe a slight increase. This might be explained by the fact that this loss of interaction contributes to higher activity by leading to faster dissociation of the enzyme-substrate complex i.e. faster turnover without affecting the rate of cleavage per se. This is of special relevance to the Arg^759^ cleavage site since it is the first to be cleaved off and the remaining two cleavages depend on this cleavage. The faster the turnover after the first cleavage during FVIII activation, the reaction can move on to the next pair of cleavages.

The amino acids surrounding Arg^1708^ seem to be most close to an optimal peptide for FVIII protein to achieve both the maximal proteolytic activation by thrombin and maximal generation of FVIIIa. Other studies have demonstrated that cleavage at Arg^1708^ enhances the specific activity of the cofactor by several folds [21, 22]. Attempts to optimize this cleavage site resulted *in vitro* in an overall reduction of specific activity. Accordingly, the most striking observation from the modeled Arg^1708^ cleavage site peptide complex is the stable U-shaped structure formed post-insertion with a highly asymmetrical charge-wise with either arms harboring positively or negatively charged residues. These residues are in their turn completely satisfied by oppositely charged thrombin residues/atoms proximal to either arm. Moreover, this peptide exhibits an asymmetrically ordered structure. While half of it retains some amount of its ordered helicity, the other half is a completely disordered coil. Consequently, the disordered region provides the flexibility to the cleavage site while the ordered region provides stability both combining to augment the cleavage capacity at the scissile bond. Hence, any structural abnormality disturbing this arrangement would lead to a decrease in activity from an insertion point of view. Unlike the other peptides, secondary structure prediction and *ab initio* models for the O-TCS 3 variant do show some small differences from its Arg^1708^ native cleavage site cleavage site (while 18 and 15 residues each of O-TCS 1 and O-TCS 2 structurally align perfectly to their native cleavage site peptide models, only 10 out of the 18 residues of O-TCS 3 align structurally to its native cleavage site peptide model). While the O-TCS 3 variant retains the U-shape of its native Arg^**1708**^ cleavage site since it still contains a critical bend inducing proline residue, it loses out on some order on the helical part of the U shape. While this would suggest easier insertion for the O-TCS 3 variant and hence likely higher activity, we observe the reverse in the expression assays. The expression data becomes clearer when we look at the post insertion interactions of the apoenzyme complex for the O-TCS 3 variant. This variant like the O-TCS 1 variant shows a significant gain in interactions (of almost all types i.e. hydrophobic, hydrogen bonds, ionic, aromatic stacking, aromatic sulphur and cation-pi interactions, S3 Table) however with the reverse outcome. In fact the O-TCS 3 variant shows the maximum gain in interactions than as its native cleavage site (inspite of the fact that the Arg^1708^ cleavage site itself is the best stabilized with maximum interacting interface residues amongst all other native cleavage sites) compared to the other optimized sequences. Therefore unlike the O-TCS 1 variant where a more stable apoenzyme complex increases the efficiency of cleavage, the O-TCS 3 variant likely results in lower turnover of the product since the more stable apoenzyme complex lengthens the rate of activation of the FVIII molecule owing to lower dissociation rates for the product.

Several studies report striking discrepancies in FVIII:C values between the chromogenic and one-stage assays [23–27]. In this study, for the majority of variants except for O-TCS 3 and O-TCS 1/3 both assays revealed similar results. For the O-TCS 1 variant the chromogenic assay was less sensitive to changes in thrombin cleavage sites than the one-stage assay. The same phenomenon is observed for patients harboring certain missense mutations with a higher value in the clotting assay compared to the chromogenic assay [27]. The observed discrepancy can be explained based on principal differences of the assays: 1) differences in the amount of thrombin (physiological amounts vs. excess of thrombin in chromogenic assay making it less sensitive to rate of activation), 2) different dilution factor of the samples (120x) for the chromogenic assay, 40x for the one-stage clotting assay assuring the activation of all FVIII molecules present in the chromogenic assay), 3) variations and differences in incubation and detection times (longer incubation with thrombin prior to initiation of FXa generation guaranteeing fully activated FVIII vs. the short time between the generation of catalytic amounts of thrombin and a measurable clot in one stage assays [24, 25].

In conclusion, our data reveals that overall the insertion of the FVIII cleavage bond loops into the thrombin active site cleft, which eventually determines the FVIII cleavage efficiency, depends on four factors: 1) the nature of the core residues comprising the cleavage bond, 2) the interaction of the neighboring residues of each site with FVIII cleavage loop, 3) the interaction of the core and neighboring residues of the inserted FVIII cleavage loop with the thrombin cleavage cleft and 4) the relative difficulty in transitioning from the local ordered secondary structure to a more flexible disordered loop post-insertion into the thrombin active site cleft. Moreover, our data indicates that the FVIII cleavage efficiency is well optimized by the theoretically semi-optimized residues at Arg^1708^, as the replacement with consensus optimizes the insertion but decreases the overall FVIII:C activity levels suggesting that the wild type thrombin cleavage sites of FVIII are already optimized.

## Supporting information

S1 Fig Introduced amino acid substitutions by mutagenesis of thrombin cleavage sites towards Leu-Val-Pro-Arg-Gly-Ser. Substitution of residues flanking the thrombin cleavage sites at A) Arg^391^> O-TCS 1, B) Arg^759^>O-TCS 2 and C) Arg^1708^> O-TCS 3. The upper sequence corresponds to the reference sequence at each cleavage site. In the lower sequence the substituted nucleotides and amino acids are highlighted.
(DOCX)

S1 Table Oligonucleotides used for mutagenesis of thrombin cleavage sites. The desired base substitutions are indicated by the respective underlined nucleotides in the primer sequence.
(DOCX)

S2 Table Hydrogen bonds of the apoenzyme complexes. (XLSX)

S3 Table Post-insertion interactions of the apoenzymes. (XLSX)
