# Supplementary material for: An in silico and in vitro approach to elucidate the impact of residues flanking the cleavage scissile bonds of FVIII
Source: PLoS One. 2017 Jul 6;12(7):e0180456. doi: 10.1371/journal.pone.0180456 (PMC5500338; doi:10.1371/journal.pone.0180456)
Supplement: S1 Fig — Substitution of residues flanking the thrombin cleavage sites at A) Arg391> O-TCS 1, B) Arg759>O-TCS 2 and C) Arg1708> O-TCS 3. The upper sequence corresponds to the reference sequence at each cleavage site. In the lower sequence the substituted nucleotides and amino acids are highlighted. (DOCX) [file pone.0180456.s001.docx]

**S1 Fig: Introduced amino acid substitutions by mutagenesis of thrombin cleavage sites towards Leu-Val-Pro-Arg-Gly-Ser.** Substitution of residues flanking the thrombin cleavage sites at A) Arg^391^> O-TCS 1, B) Arg^759^>O-TCS 2 and C) Arg^1708^> O-TCS 3. The upper sequence corresponds to the reference sequence at each cleavage site. In the lower sequence the substituted nucleotides and amino acids are highlighted.

**
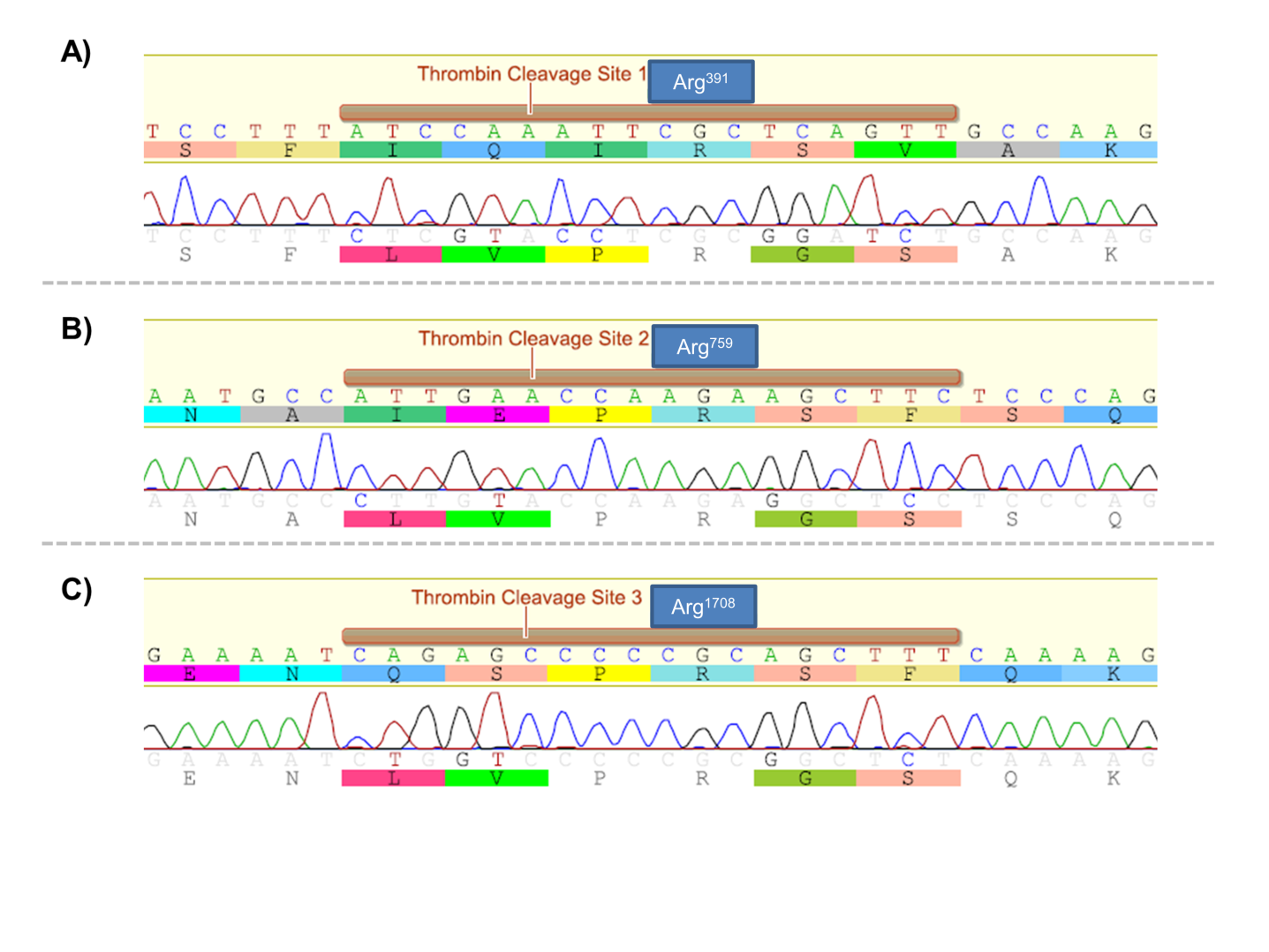
**
