# Supplementary material for: An in silico and in vitro approach to elucidate the impact of residues flanking the cleavage scissile bonds of FVIII
Source: PLoS One. 2017 Jul 6;12(7):e0180456. doi: 10.1371/journal.pone.0180456 (PMC5500338; doi:10.1371/journal.pone.0180456)
Supplement: S1 Table — The desired base substitutions are indicated by the respective underlined nucleotides in the primer sequence. (DOCX) [file pone.0180456.s002.docx]

**S1 Table. Oligonucleotides used for mutagenesis of thrombin cleavage sites**. The desired base substitutions are indicated by the respective underlined nucleotides in the primer sequence**.**

| **Primer** | **Sequence 5´🡪3´** |
| --- | --- |
| 388-393 MP | AT GAC AAC TCT CCT TCC TTT CTC GTA CCT CGC GGA TCT GCC AAG AAG CAT CCT AAA AC |
| 756 Ile>Leu, 757 Glu>Val-F | GT AAA AAC AAT GCC CTT GTA CCA AGA AGC TTC |
| 756 Ile>Leu, 757 Glu>Val-R | TGG TAC AAG GGC ATT GTT TTT ACT CAG CAA G |
| 760 Ser>Gly, 761 Phe>Ser-F | GTA CCA AGA GGC TCC TCC CAG AAT TC |
| 760 Ser>Gly, 761 Phe>Ser-R | GA GGA GCC TCT TGG TAC AAG GGC ATT G |
| 1705 Glu>Leu, 1706 Ser>Val-F | GAA AAT CTG GTC CCC CGC AGC |
| 1705 Glu>Leu, 1706 Ser>Val-R | G GAC CAG ATT TTC ATC CTC ATC ATA AAT G |
| 1709 Ser>Gly, 1710 Phe>Ser-F | CGC GGC TCT CAA AAG AAA ACA CG |
| 1709 Ser>Gly, 1710 Phe>Ser-R | G AGA GCC GCG GGG GAC CAG ATT T |

MP, megaprimer; F, forward primer; R, reverse primer
